# Identification of Differentially Expressed Genes and Protein-Protein Interaction in Patients With COVID-19 and Diabetes Peripheral Neuropathy: A Bioinformatics and System Biology Approach

**DOI:** 10.7759/cureus.58548

**Published:** 2024-04-18

**Authors:** Fahim Alam Nobel, Mohammad Kamruzzaman, Mohammad Asaduzzaman, Mohammad Nasir Uddin, Hasib Ahammad, Mehedi Mahmudul Hasan, Tanu Rani Kar, Farha Matin Juliana, Golap Babu, Mohammod Johirul Islam

**Affiliations:** 1 Biochemistry and Molecular Biology, Mawlana Bhashani Science and Technology University, Tangail, BGD; 2 Biochemistry and Molecular Biology, Noakhali Science and Technology University, Noakhali, BGD; 3 Fisheries and Marine Science, Noakhali Science and Technology University, Noakhali, BGD; 4 Biochemistry and Molecular Biology, Primeasia University, Dhaka, BGD; 5 Biochemistry and Molecular Biology, Jahangirnagar University, Savar, Dhaka, BGD

**Keywords:** limma, protein-protein interaction network, therapy, gene ontology, functional enrichment, differentially expressed genes (degs), diabetes peripheral neuropathy, diabetes, covid-19, sars-cov-2

## Abstract

The coronavirus disease 2019 (COVID-19) pandemic has had a significant impact globally, resulting in a higher death toll and persistent health issues for survivors, particularly those with pre-existing medical conditions. Numerous studies have demonstrated a strong correlation between catastrophic COVID-19 results and diabetes. To gain deeper insights, we analysed the transcriptome dataset from COVID-19 and diabetic peripheral neuropathic patients. Using the R programming language, differentially expressed genes (DEGs) were identified and classified based on up and down regulations. The overlaps of DEGs were then explored between these groups. Functional annotation of those common DEGs was performed using Gene Ontology (GO), Kyoto Encyclopedia of Genes and Genomes (KEGG), Bio-Planet, Reactome, and Wiki pathways. A protein-protein interaction (PPI) network was created with bioinformatics tools to understand molecular interactions. Through topological analysis of the PPI network, we determined hub gene modules and explored gene regulatory networks (GRN). Furthermore, the study extended to suggesting potential drug molecules for the identified mutual DEG based on the comprehensive analysis. These approaches may contribute to understanding the molecular intricacies of COVID-19 in diabetic peripheral neuropathy patients through insights into potential therapeutic interventions.

## Introduction

Following the December 2019 outbreak in China, coronavirus disease 2019 (COVID-19) was confirmed as a new type of coronavirus in early 2020 . The infection is caused by a virus of the coronaviridae family termed severe acute respiratory syndrome coronavirus 2 (SARS-CoV-2) [1]. This deadly virus uses human angiotensin-converting enzyme-2 (ACE-2) receptors to enter the human body [2]. The virus is primarily transmitted via respiratory droplets from one person to another [3]. According to the statistics on the worldmeter website, as of April 2024, there were 704,753,890 confirmed COVID-19 cases and 7,010,681 mortalities worldwide. 

Diabetes often results from insufficient insulin production by the pancreas or an inadequate cellular response to the insulin produced [4]. This can lead to elevated blood glucose levels. When individuals with diabetes contract a viral infection, their recovery may be more challenging due to fluctuating blood glucose levels and the presence of diabetic-related conditions. The International Diabetes Federation (IDF) identifies two key factors contributing to this increased complexity. First, the compromised immune system makes it harder to combat viruses, potentially prolonging recovery. Second, the virus may thrive in an environment with elevated blood glucose levels.

Peripheral neuropathy, a common complication of diabetes, results in damage to peripheral nerves, leading to sensory disturbances, pain, and motor deficits. Long-term diabetic patients with associated comorbidities have been observed to experience a more acute form of COVID-19 compared to non-diabetic individuals [5-6]. The interplay between hyperglycemia and hyper-inflammation related to COVID-19 may render diabetic patients more vulnerable, potentially increasing their fragility and mortality during the SARS-CoV-2 infection [7-8]. Given these factors, it has been suggested that COVID-19 and diabetes may exhibit various pathological interactions. Therefore, it is crucial to investigate their molecular relationship. In this study, we investigated the large-scale transcriptomic data of COVID-19 and diabetic patients suffering from the complications of diabetic peripheral neuropathy. This is the first time we have studied the transcriptomics of patients with diabetic neuropathic complications and COVID-19 infection. So, to expose the key fact, two datasets (GSE 147507 and GSE 95849) were selected for the transcriptomic level study. Understanding the molecular pathways involved is critical for developing therapeutic strategies or repurposing existing medications for COVID-19-infected diabetic patients with peripheral neuropathy.

In our research, we employed bioinformatics and systems biology approaches to identify differentially expressed genes (DEGs) from two datasets: GSE 147507 (comprising human COVID-19 samples, including controls) and GSE 95849 (derived from diabetic patients with peripheral neuropathy). After identifying DEGs, we focused on mutual DEGs shared between these datasets. Subsequently, we delved into gene ontology, informative pathways, protein-protein interaction networks, hub genes, modules, and transcription factor (TF)-miRNA network analyses using these mutual DEGs. Finally, considering all relevant factors, we proposed a suitable drug molecule.

## Materials and methods

In-silico analysis

**Figure 1:**
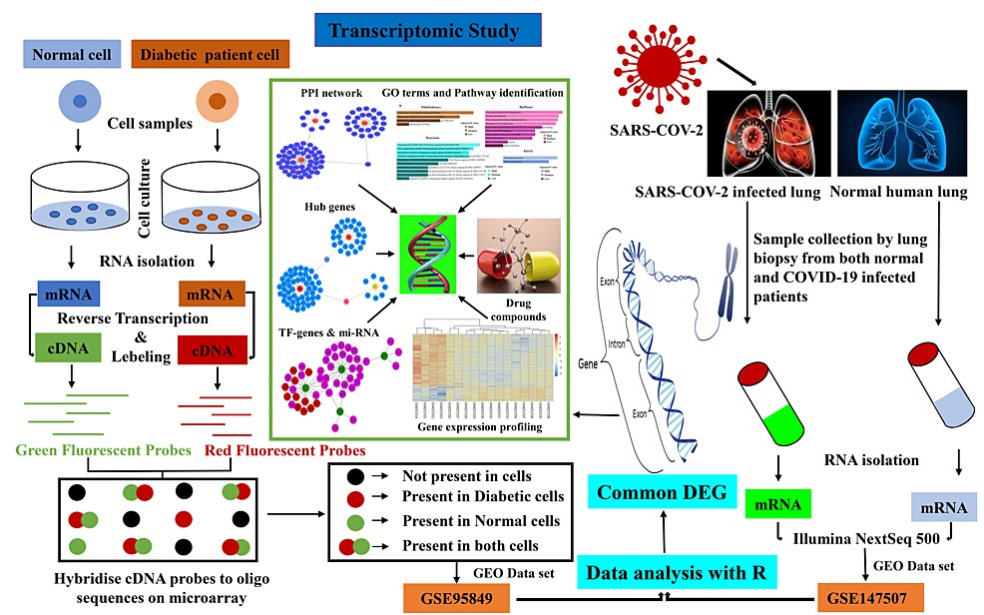
Schematic workflow representing overall in-silico analysis The microarray (GSE95849) and RNA seq (GSE147507) data were retrieved from the National Center for Biotechnology Information (NCBI) Gene Expression Omnibus (GEO) data set. The data set was then individually analysed through the R programming language to determine similar differentially expressed genes (DEGs). After analysis, the common DEGs were set for transcriptomic study. The transcriptomic study included (protein-protein interaction (PPI), hub genes, transcription factor (TF)-miRNA, gene ontology (GO) terms, pathway identification, gene expression profiling, and the generation of drug compounds).

The entire research process is visually summarized in Figure 1.

Data collection

The National Center for Biotechnology (NCBI) Gene Expression Omnibus (GEO) database has been utilised to investigate the genetic associations between SARS-CoV-2 and diabetic related disorders [9]. To simulate SARS-CoV-2 infection, the GSE147507 dataset was studied [10]. From 110 samples, 23 data on COVID-19-infected human cells were taken as diseased, and 22 data of mock and healthy lung biopsy samples were treated as control patients. Another dataset, GSE95849, focused on transcriptional profiling of diabetic peripheral neuropathy patients, diabetic patients, and healthy participants [11]. In this dataset, 12 samples were designated as diseased, and six were considered control patients.

Identification of DEGs

The popular and widely used limma package [12] of the R programming language was utilized to find the differentially expressed genes (DEGs) individually for GSE147507 and GSE95849 datasets. The cut-off was set at 0.05 for the adjusted P-value to identify the relevant genes. Following identification, the common DEGs between the two datasets were computed using the intercept functions of the R programming language.

Gene ontology (GO) and pathway-based analysis

Gene set enrichment analysis is a computational and statistical approach that investigates a set of genes' biological, molecular, and cellular features (collectively known as GO) and their cell informative pathways [13]. GO and pathway-based analysis are required to comprehend the biological implications of DEGs. For GO and route enrichment analyses, a web-based program called EnrichR (https://maayanlab.cloud/Enrichr/) was used [14]. We utilized the WikiPathways [15], Kyoto Encyclopedia of Genes and Genomes (KEGG) [16], Bioplanet [17] and Reactome [18] databases from EnrichR, which has 102 extensive collections of libraries. The adjusted P-value of 0.05 was set as a standard value for quantifying the most significant listed GO and pathways for common DEGs. WikiPathways was launched in 2008, and it acts as a good platform for biological knowledge in the form of pathway diagrams [19]. KEGG is a manually edited database resource (https://www.kegg.jp) that integrates biological objects classified into systems, genetic, chemical, and health information. More features were added to facilitate a more profound comprehension of more fundamental issues, such as how molecular network systems originated in cells, and co-evolved with the genome. They were passed on to the current species [20]. The Reactome is a knowledge base comprehensive database tool for discovering functional relationships in biological data and provides the molecular level of multiple cellular processes [21].

Identification of protein-protein interaction network analysis

The inspection and characterization of the PPI network are the primary goals in cellular and systems biology for understanding and learning about cellular machinery activities [22-24]. To represent functional and physical interaction, a protein-protein interaction network of common DEGs was generated using the IMEx Interactome database of Network Analyst (https://www.networkanalyst.ca/) platform with a default cut-off score of 900 [25]. Usually, a confidence score of 900 is considered high and indicates that the reported interactions are highly reliable. After network generation, we visualized the PPI interaction network with the Cytoscape software version v3.8.2 (https://cytoscape.org/). It is a free-source software in which multiple datasets are aggregated to enhance performance for various interactions like PPIs, genetic interactions, protein-DNA interactions, and many more [26].

Determination of hub genes and submodules network

Hub genes are essential to the upkeep of a biological network's connection and functionality, such as a network regulating genes or one involving interactions between proteins. Identifying the hub genes is crucial to comprehending how biological systems have been organised and regulated. The hub genes in this study were detected using the Cytoscape plugin cytoHubba (http://apps.cytoscape.org/apps/cytohubba). Cytohubba has 11 topological techniques for managing network nodes. "Modules" are the sites where the hub genes are tightly integrated into the PPI network. ClusterViz (https://apps.cytoscape.org/apps/clusterviz), a Cytoscape plugin, is employed for module analysis in the existing network.

Recognition of the TF-miRNA co-regulatory network

The most critical factor in regulating gene expression is the intricate regulatory interactions among transcription factors (TFs), microRNAs (miRNAs), and differentially expressed genes (DEGs). These TF-miRNAs influenced DEGs at both the transcriptional and posttranscriptional stages. Understanding these mechanisms is essential for distinguishing between healthy cellular activities and disease situations. RegNetwork repository database from the Network Analyst platform (https://www.networkanalyst.ca/) has been chosen to identify the TF-miRNA network. The network is visualized using the Cytoscape program (https://cytoscape.org/).

The prediction of therapeutic drug compounds

For COVID-19 individuals with diabetes and peripheral neuropathy, drug assessment is crucial. The Enrichr platform's Drug Signatures Database (DSigDB) identifies medications for this ailment. The database has 22 527 gene sets, 19 531 genes, and 17 389 distinct chemicals. Drugs having an adjusted P value of less than 0.05 were considered viable treatments for the ailment.

## Results

Determination of DEGs in COVID-19 and diabetic comorbidities

**Figure 2:**
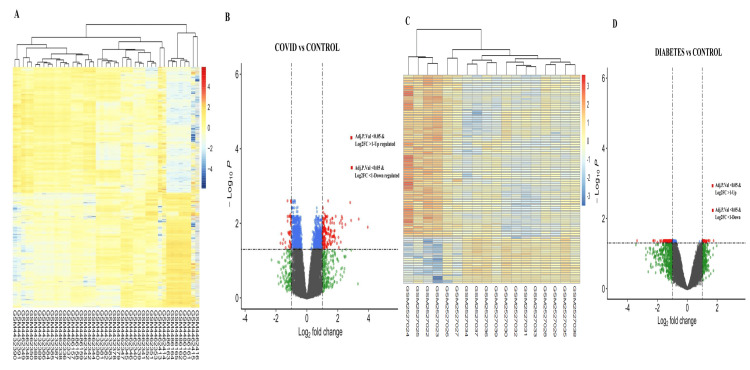
Gene expression profiling of common DEGs and their regulation (upregulation and downregulation) (A), (C) A heatmap shows the expression level of similar DEGs from GSE147507 and GSE95849 datasets. (B), (D) A volcano plot visualizes the upregulated and downregulated common DEGs of GSE147507 and GSE95849 datasets.

We identified 1039 genes (38 genes were down-regulated and 901 genes were up-regulated) expressed differentially in COVID-19-infected patients compared to controls (based on the adjusted P-value of 0.05) (Figure 2). On the other hand, 117 genes were differentially expressed in diabetes and diabetic peripheral neuropathic patients compared to controls (93 genes were downregulated and 23 were upregulated) (Figure 2).

Exploration of common DEG between COVID-19 and diabetic complications

**Figure 3:**
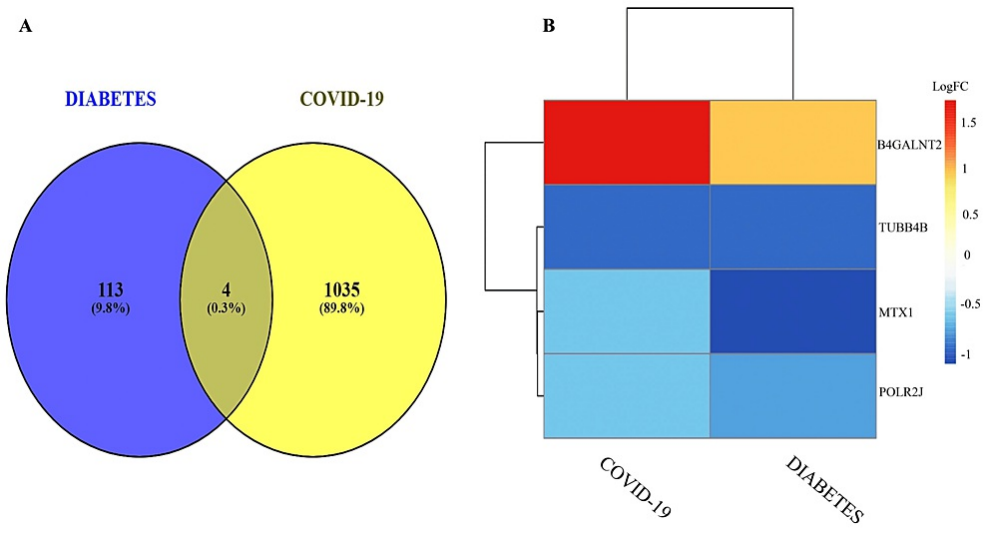
Common DEGs between COVID-19 and diabetic complications (A) Identification of common DEGs from GSE147507 and GSE95849 datasets through the Venn diagram. (B) The expression level of common DEGs in GSE147507 (COVID-19) and GSE95849 (diabetic peripheral neuropathy) datasets. From the heat map, it was observed that B4GALNT2 gene expression is upregulated in COVID-19 than in diabetics. TUBB4B expression is the same in both COVID-19 and diabetic patients. On the other hand, MTX1 and POLR2J are downregulated and lowly expressed in diabetic patients than COVID-19.

The identification of common DEGs is a critical component of transcriptomics research. We found four genes commonly expressed in these two separate situations (Figure 3A). Figure 3B illustrates a heatmap of those four common genes (B4GALNT2, MTX1, POLR2J, TUBB4B) with their expression parameter logFC.

**Figure 4:**
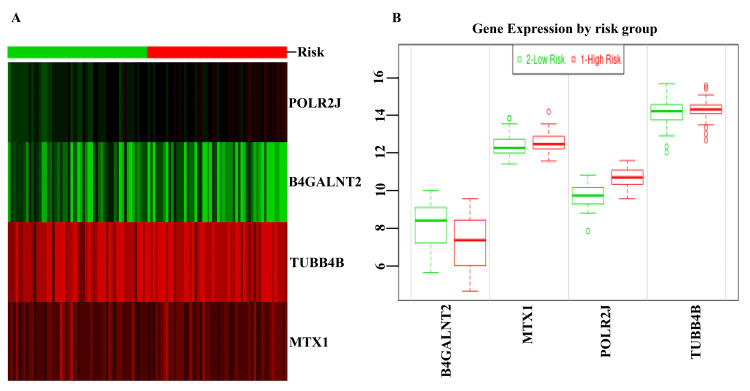
Risk group identification and comparison (A) Risk group identification of common DEGs. (B) Comparisons of risk groups in the form of a box plot.

The validation and the verification were confirmed according to the risk category (Figure 4A). The heatmap indicated that the MTX1 and TUBB4B genes are highly prone to inflammation. The identical circumstances are shown in Figure 4B as well.

GO and cell signalling pathway enrichment analysis

GO

Table 1 shows that the gene B4GALNT2 is involved in most of the four biological processes. The POLR2J gene was highly enhanced in the negative regulation of DNA recombination at the telomere, regulation of DNA recombination at the telomere, and positive regulation of viral transcription. The gene TUBB4B is involved in natural killer cell-mediated cytotoxicity (Table 1).

**Table 1:** Integration of top GO term related to mutual DEGs GO: Gene ontology, DEG: Differentially expressed gene, UDP: Uridine diphosphate, LRR: Leucine rich repeat, MHC: Major histocompatibility complex, GTP: Guanosine triphosphate, MIB: Mitochondrial intermembrane space bridging, SAM: Sterile alpha motif

GO biological process	GO ID	Term	Genes
	GO:0006040	amino sugar metabolic process	B4GALNT2
	GO:0009225	nucleotide-sugar metabolic process	B4GALNT2
	GO:0006047	UDP-N-acetylglucosamine metabolic process	B4GALNT2
	GO:0002228	natural killer cell-mediated immunity	TUBB4B
	GO:0048239	negative regulation of DNA recombination at telomere	POLR2J
	GO:0072695	regulation of DNA recombination at telomere	POLR2J
	GO:0042267	natural killer cell-mediated cytotoxicity	TUBB4B
	GO:0009312	oligosaccharide biosynthetic process	B4GALNT2
	GO:0016051	carbohydrate biosynthetic process	B4GALNT2
	GO:0050434	positive regulation of viral transcription	POLR2J
GO molecular function	GO:0030275	LRR domain binding	POLR2J
	GO:0042288	MHC class I protein binding	TUBB4B
	GO:0008376	acetylgalactosaminyltransferase activity	B4GALNT2
	GO:0042287	MHC protein binding	TUBB4B
	GO:0003899	DNA-directed 5'-3' RNA polymerase activity	POLR2J
	GO:0034062	5'-3' RNA polymerase activity	POLR2J
	GO:0003725	double-stranded RNA binding	TUBB4B
	GO:0008194	UDP-glycosyltransferase activity	B4GALNT2
	GO:0016758	hexosyltransferase activity	B4GALNT2
	GO:0005525	GTP binding	TUBB4B
GO cellular component	GO:0140275	MIB complex	MTX1
	GO:0001401	SAM complex	MTX1
	GO:0005665	RNA polymerase II, core complex	POLR2J
	GO:0005742	mitochondrial outer membrane translocase complex	MTX1
	GO:0031305	integral component of the mitochondrial inner membrane	MTX1
	GO:0065010	extracellular membrane-bounded organelle	TUBB4B
	GO:1903561	extracellular vesicle	TUBB4B
	GO:0035578	azurophil granule lumen	TUBB4B

**Figure 5:**
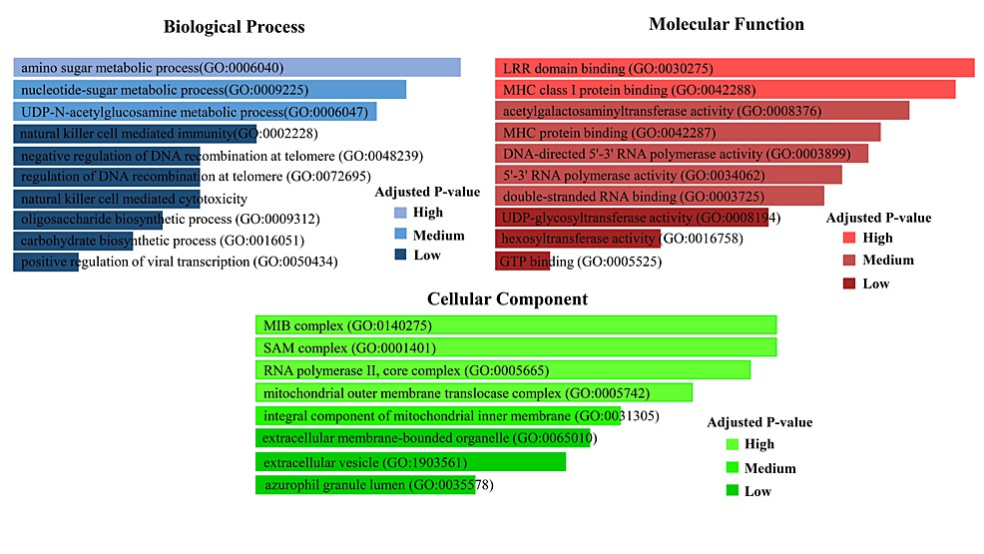
Top biological, molecular, and cellular functions of mutual DEGs according to the adjusted P-value DEG: Differentially expressed gene

POLR2J expression significantly impacts leucine rich repeat (LRR) domain binding, DNA-directed 5'-3' RNA polymerase activity, and 5'-3' RNA polymerase activity (depending on its equal significance value). The genes TUBB4B and B4GALNT2 have implications in major histocompatibility complex (MHC) class I protein binding, MHC protein binding, and acetylgalactosaminyltransferase activity (Table 1). Both have also performed double-stranded RNA binding, guanosine triphosphate (GTP) binding, uridine diphosphate (UDP)-glycosyltransferase activity, and hexosyltransferase activity. The significant influence on cellular components reveals that the MTX1 gene had great functionality on the MIB complex, SAM complex, mitochondrial outer membrane translocase complex, and an integral component of the mitochondrial inner membrane (Table 1). Furthermore, TUBB4B was found in extracellular membrane-bounded organelles, extracellular vesicles, azurophil granule lumen, and POLR2J in forming the RNA polymerase II core complex (Table 1). The representation of the GO term is also summarized in the form of a linear bar diagram in Figure 5, where different color intensities denote the DEG functionality.

Pathway analysis

We combined several pathways related to the common DEGs from COVID-19, diabetes, and diabetes peripheral neuropathy patients and categorized them according to their adjusted P value of 0.05 (Table 2). Four pathway activities have been identified through wiki pathways, where POLR2J is involved in Eukaryotic Transcription Initiation WP405 and Pyrimidine metabolism WP4022. Meanwhile, the TUBB4B gene activates the pathogenic *Escherichia coli* infection WP2272 and the Parkin-Ubiquitin Proteasomal System pathway WP2359. Rectome, a widely used and verified database, showed pathways related to the genes POLR2J and TUBB4B. The database displayed seven pathways, including signaling by FGFR2 IIIa TM Homo sapiens R-HSA-8851708, MicroRNA (miRNA) biogenesis in Homo sapiens R-HSA-203927, Abortive elongation of HIV-1 transcript in the absence of Tat Homo sapiens R-HSA-167242, FGFR2 alternative splicing Homo sapiens R-HSA-6803529, RNA Pol II CTD phosphorylation and interaction with CE Homo sapiens R-HSA-167160, RNA Pol II CTD phosphorylation and interaction with CE Homo sapiens R-HSA-77075, PIWI-interacting RNA (piRNA) biogenesis Homo sapiens R-HSA-5601884 and three pathways like Post-chaperonin tubulin folding pathway Homo sapiens R-HSA-389977, the formation of tubulin folding intermediates by CCT/TriC Homo sapiens R-HSA-389960, and Prefoldin mediated transfer of substrate to CCT/TriC Homo sapiens R-HSA-389957 has been activated by POLR2J and TUBB4B respectively. Another database, Bio Planet explored seven different pathways influenced by gene POLR2J, and they were Viral messenger RNA biosynthesis, Regulatory RNA pathways, Messenger RNA capping, a Dual incision reaction in TC-NER, RNA polymerase, HIV-1 transcription initiation, and Eukaryotic transcription initiation. This database has also shared the activation of the post-chaperonin tubulin folding pathway, the Cooperation of prefoldin and TriC/CCT in actin, and the tubulin folding pathway regulated by the TUBB4B gene. A single pathway in the Bio Planet database called Protein metabolism strongly mediated MTX1 and TUBB4B (Table 2).

**Table 2:** Top pathway analysis based on adjusted P value of shared DEGs DEG: Differentially expressed gene

Databases	Pathways	Genes
WikiPathways	Eukaryotic Transcription Initiation WP405	POLR2J
	Pathogenic Escherichia coli infection WP2272	TUBB4B
	Parkin-Ubiquitin Proteasomal System pathway WP2359	TUBB4B
	Pyrimidine metabolism WP4022	POLR2J
Reactome	Signaling by FGFR2 IIIa TM Homo sapiens R-HSA-8851708	POLR2J
	Post-chaperonin tubulin folding pathway Homo sapiens R-HSA-389977	TUBB4B
	MicroRNA (miRNA) biogenesis Homo sapiens R-HSA-203927	POLR2J
	Abortive elongation of HIV-1 transcript in the absence of Tat Homo sapiens R-HSA-167242	POLR2J
	Formation of tubulin folding intermediates by CCT/TriC Homo sapiens R-HSA-389960	TUBB4B
	FGFR2 alternative splicing Homo sapiens R-HSA-6803529	POLR2J
	Prefoldin mediated transfer of substrate to CCT/TriC Homo sapiens R-HSA-389957	TUBB4B
	RNA Pol II CTD phosphorylation and interaction with CE Homo sapiens R-HSA-167160	POLR2J
	RNA Pol II CTD phosphorylation and interaction with CE Homo sapiens R-HSA-77075	POLR2J
	PIWI-interacting RNA (piRNA) biogenesis Homo sapiens R-HSA-5601884	POLR2J
BioPlanet	Viral messenger RNA biosynthesis	POLR2J
	Protein metabolism	MTX1; TUBB4B
	Post-chaperonin tubulin folding pathway	TUBB4B
	Regulatory RNA pathways	POLR2J
	Cooperation of prefoldin and TriC/CCT in actin and tubulin folding	TUBB4B
	Messenger RNA capping	POLR2J
	Dual incision reaction in TC-NER	POLR2J
	RNA polymerase	POLR2J
	HIV-1 transcription initiation	POLR2J
	Eukaryotic transcription initiation	POLR2J
KEGG	Huntington disease	TUBB4B; POLR2J
	RNA polymerase	POLR2J

**Figure 6:**
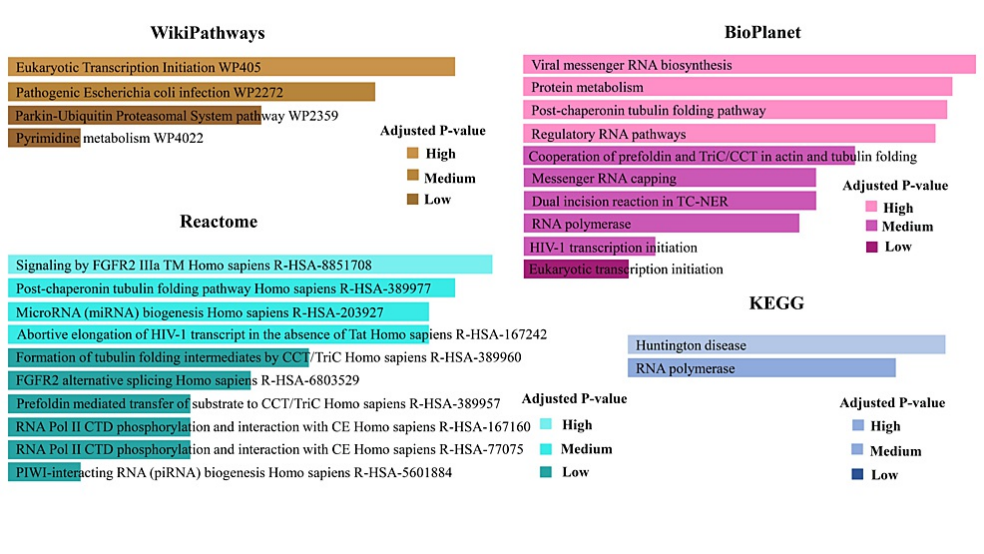
Top pathway identification (Wiki Pathways, BioPlanet, Reactome, KEGG) based on adjusted P-value. KEGG: Kyoto Encyclopedia of Genes and Genomes

A KEGG database showed the activity of two DEGs (TUBB4B, POLR2J) (Table 2). Both TUBB4B POLR2J genes worked on Huntington's disease and RNA polymerase (Table 2). The pathways related to the common DEGs are also shown in Figure 6.

Protein-protein interaction network analysis of common DEG

**Figure 7:**
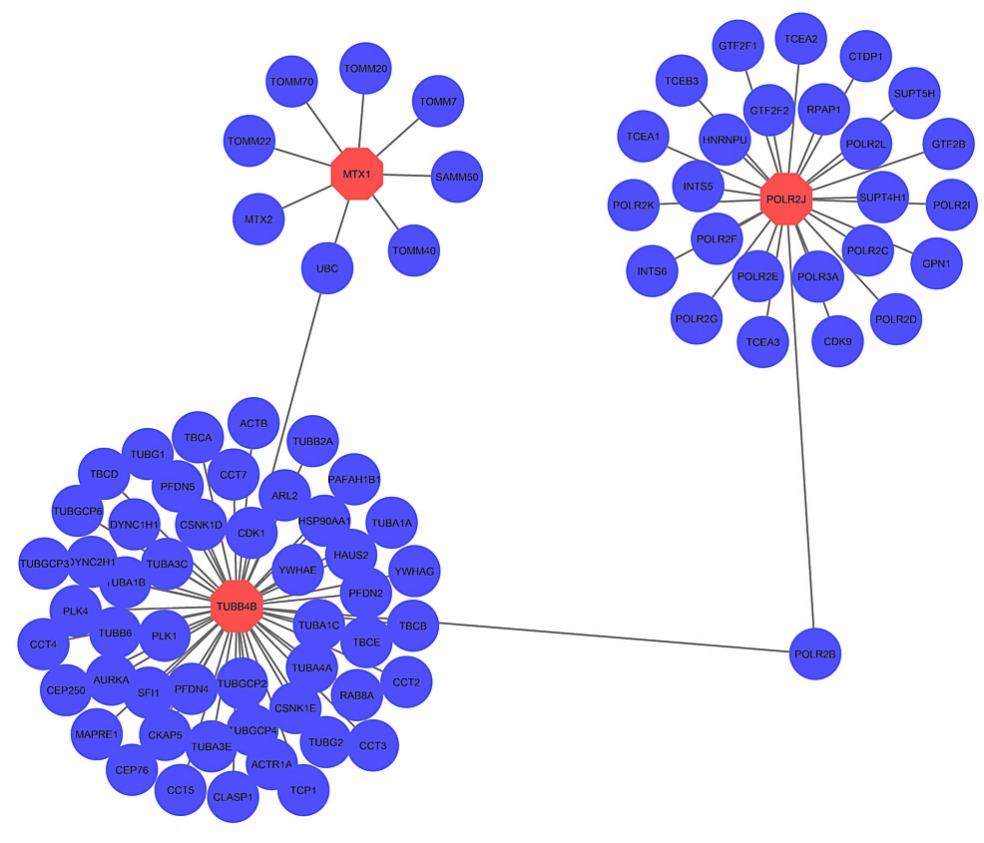
Protein-protein interaction network analysis of common DEGs determined in this study Protein-protein interaction network analysis of common differentially expressed genes (DEGs) determined from coronavirus disease 2019 (COVID-19), diabetic as well as diabetic peripheral neuropathy affected patient datasets. The common genes are displayed through pink color, while the light blue color dictates, the genes that have a strong connection with common genes. The network is formed by 86 nodes with 87 edges.

After enrichment analysis, the common DEGs have been subjected to the Network Analyst platform to view the interaction profile among the other genes. From there, we identified the relationships of several genes with the MTX1, POLR2J, and TUBB4B genes (Figure 7).

TUBB4B and POLR2J interact with 53 and 26 genes, respectively, whereas MTX1 interacts with only eight genes (Figure 7). There was no interaction found for B4GALNT2. 87 nodes with 86 edges were formed in this PPI network (Figure 7).

Detection of hub genes based on topological analysis and module identification from the PPI network

To achieve the central gene, the degree of the topological algorithm was utilized, and it revealed five genes (MTX1, POLR2J, POLR2B, UBC, and TUBB4B) (Table 3).

**Table 3:** Topological results analysis for top five hub genes

Hub gene	Degree	Bottle Neck	Closeness centrality	Betweenness centrality	Stress
UBC	2	9	37.58333	1248	1248
POLR2B	2	87	42.08333	3120	3120
MTX1	8	8	30.75	1148	1148
POLR2J	26	87	45.15	3650	3650
TUBB4B	52	87	63.66667	6536	6536

**Figure 8:**
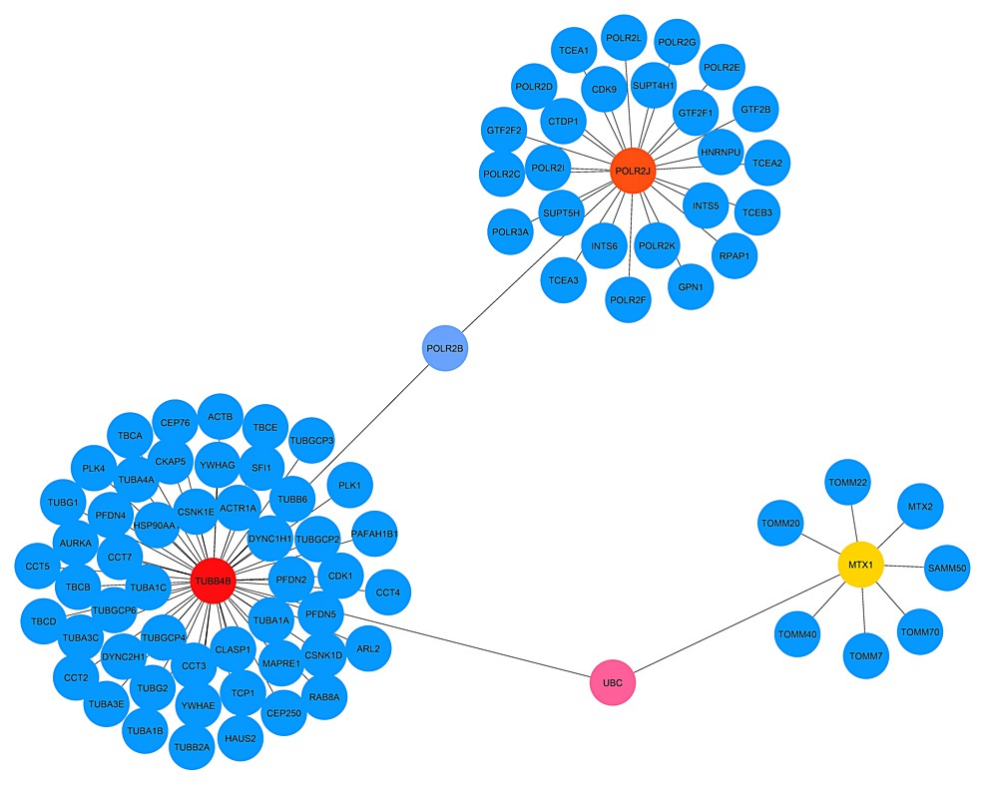
Generation of hub genes from the PPI network MTX1(yellow), UBC (pink), POLR2J(orange), and TUBB4B(red) are the hub genes in the network. The network is determined by 87 nodes and 86 edges.

These five genes were highly interconnected to each other and were termed hub genes (Figure 8).

**Figure 9:**
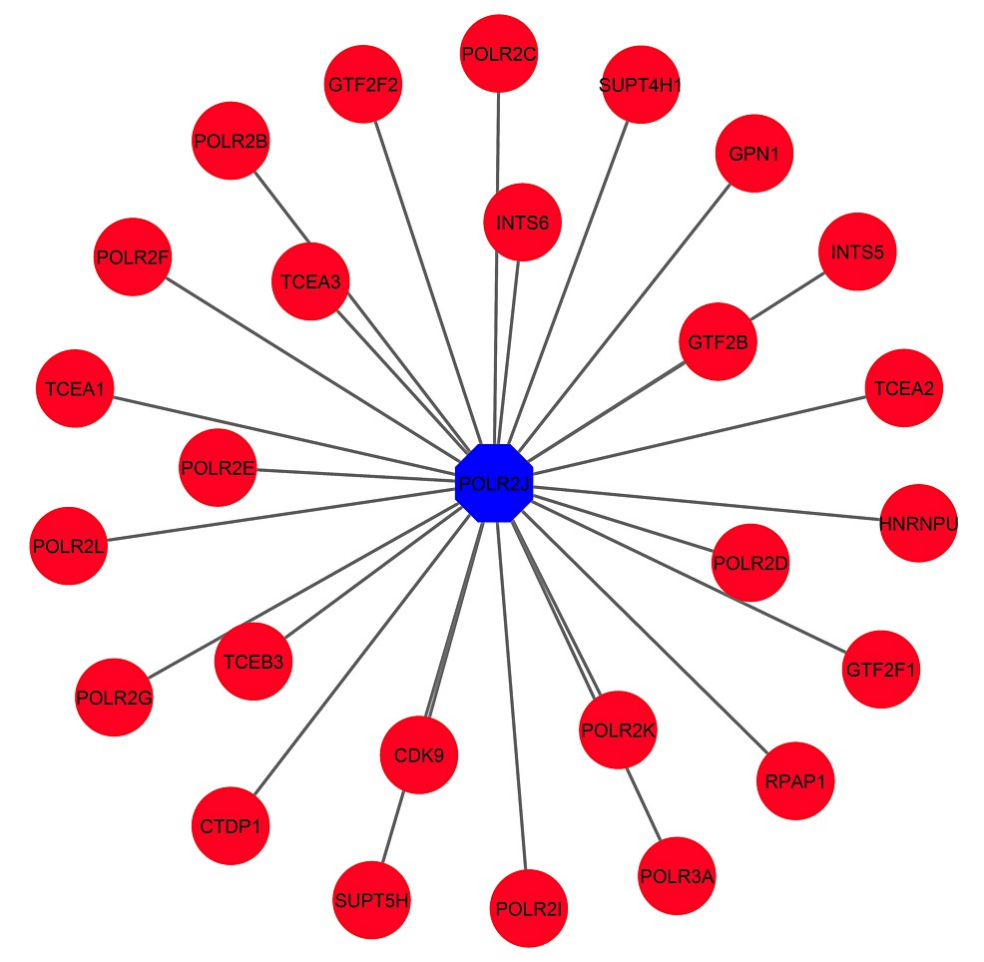
Module interaction network analysis to show the highly interconnected hub genes with their related ones Highly interconnected hub genes (blue) and their related ones (red).

It is essential to identify the hub genes because they could be potential biomarkers for future therapy in many diseases. We also tried to identify module network analysis to see the close connectivity among genes, from where we found only one interconnected sub-module network consisting of 27 nodes with 26 edges (Figure 9).

Gene regulatory network (GRN) analysis of common DEG

**Figure 10:**
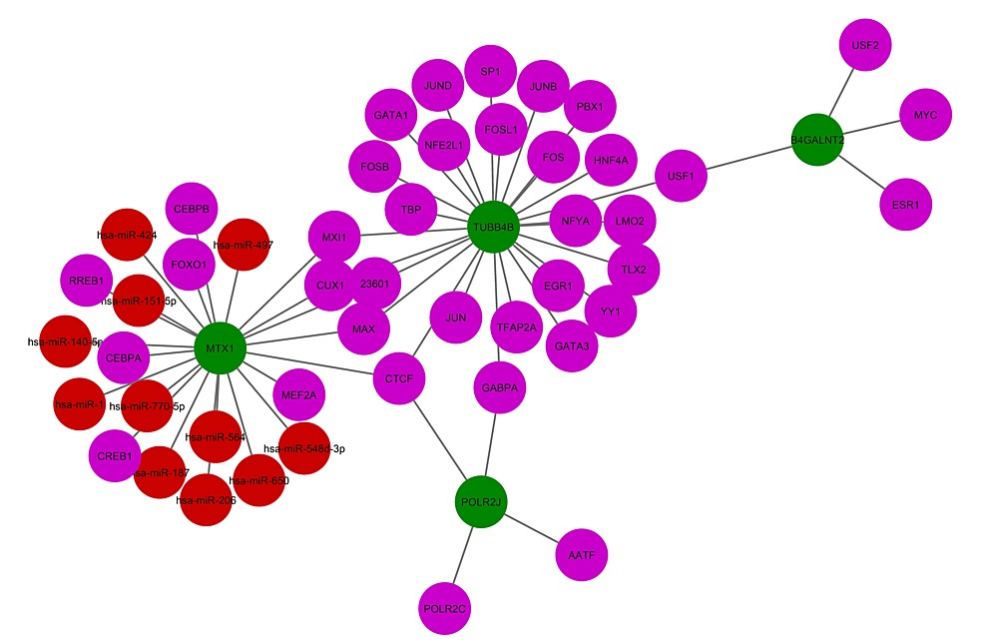
TF and miRNA interaction network analysis The network consists of 53 nodes and 57 edges. The miRNA network is shown in a red circle, while the transcription factor (TF) is demonstrated through a violet color.  The network is linked through green circles denoted as central nodes.

To get inside the common DEGs, studies of TF and miRNA are essential. Because the majority of genes are regulated at both the transcriptional (via TF) and post-transcriptional (via miRNA) levels, both TF and miRNA have highly significant molecular insights. In this study, we scrutinized 37 TF and 11 miRNA highly connected by the existing common differentially expressed genes of COVID-19 and diabetic patients having complications with diabetic peripheral neuropathy (Figure 10).

Repurposing traditional drug compounds

**Figure 11:**
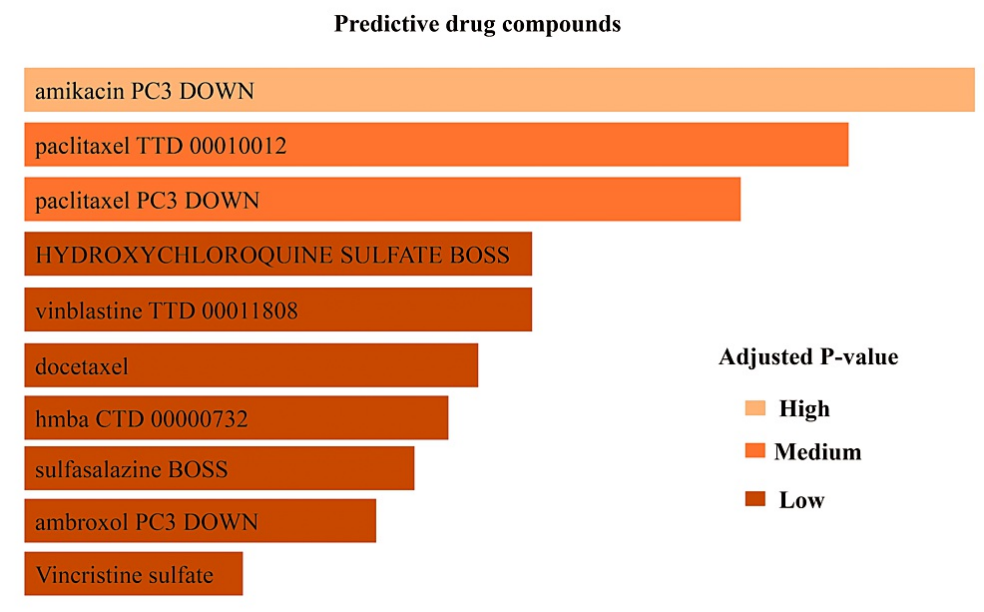
Drug compounds for healing COVID-19-affected and diabetic peripheral neuropathic patients COVID-19: Coronavirus disease 2019

Table 4 represents drug molecules that are closely related to shared genes. The drugs that were involved here are amikacin PC3 DOWN, paclitaxel TTD 00010012, paclitaxel PC3 DOWN, hydroxychloroquine sulfate, docetaxel, hmba CTD 00000732, sulfasalazine BOSS, ambroxol PC3 DOWN, vincristine sulfate. The potency of these drugs is also shown in Figure 11.

**Table 4:** Determination of therapeutic compounds relying on COVID-19 and diabetic complications COVID-19: Coronavirus disease 2019

Name of drugs	Genes
amikacin PC3 DOWN	TUBB4B; POLR2J
paclitaxel TTD 00010012	TUBB4B
paclitaxel PC3 DOWN	TUBB4B; POLR2J
HYDROXYCHLOROQUINE SULFATE BOSS	MTX1
vinblastine TTD 00011808	TUBB4B
Docetaxel	TUBB4B
hmba CTD 00000732	TUBB4B
sulfasalazine BOSS	MTX1
ambroxol PC3 DOWN	TUBB4B; POLR2J
Vincristine sulfate	TUBB4B

## Discussion

The progression of SARS-CoV-2 infection might vary across individuals, with the genetic component of an individual having a crucial impact. Determining the genes linked to different levels of COVID-19 severity can be helpful in therapeutic settings. Gaining insights into the primary genes associated with severe disease manifestations is essential for predicting the most harmful symptoms and responding promptly to patients in this context.

Diabetic peripheral neuropathy is one of the critical complications of diabetes, affecting millions of people worldwide [27]. This complication affects more than half of diabetic patients [28]. In this study, we focused on the genes that are mutually expressed in both conditions. By analysing two datasets, four DEGs were found to be commonly expressed. Among them, B4GALNT2 was highly expressed in COVID-19 as well as diabetic peripheral neuropathic patients (Figure 3). Conversely, the remaining three genes showed downregulation in both cases. Understanding the ontological and pathway processes of the DEG offers valuable insights into the severity of COVID-19 and its comorbidities. The significant functionality of the common DEGs is shown in Table 1, where some functionalities are more prevalent and related to disease severity. On the other hand, Table 2 represents the principal pathways related to the identified genes. A visual representation of the gene ontology (GO) terms and the pathways is shown in Figure 5.

A strong PPI, gene-miRNA, and TF-gene regulatory network was generated based on the common DEGs. The PPI network was constructed by meticulously connecting 87 nodes via 86 edges (Figure 7). This analysis brought to light five central nodes or hub genes (MTX1, POLR2J, TUBB4B, POLR2B, and UBC) that govern all the interactions within the network (Figure 8). After hub gene generation, highly interconnected modules were also determined to have the most promising interaction network (Figure 9). Transcriptional factors (TF) and post-transcriptional regulation (miRNA) are the two essential components of gene expression. Here, TF and miRNA for common DEG were also determined to understand what types of regulatory genes are directly involved in the network. In total, 37 TFs and 11 miRNAs were identified through this interacting network (Figure 10). The majority of the TFs interacted strongly with TUBB4B genes. On the other hand, miRNA was only associated with MTX1 genes (Figure 10). This type of network has a potential implication in many research projects concerning the prediction of disease genes, taking into account factors such as disease loci, gene-disease phenotypic relationships, and disease-specific changes in gene expression [29]. Using DSigDB, the top 10 therapeutic interventions targeting the DEG were identified (Table 4). Among them, amikacin PC3 DOWN, paclitaxel PC3 DOWN, and ambroxol PC3 DOWN can inhibit the expression of 2 genes (TUBB4B and POLR2J). Only TUBB4B can be controlled by paclitaxel TTD 00010012, docetaxel, hmba CTD 00000732, and vincristine sulfate. Hydroxychloroquine sulfate Boss and the sulfasalazine Boss can regulate the MTX1 gene. No therapy was identified against the B4GALNT2 genes (Table 4). Moreover, no interaction network and pathways were found to be activated for that gene (Figures 7-8).

So, with other common DEG, the gene (B4GALNT2) can be a potential biomarker for revealing the connection between COVID-19 and associated comorbidities. For instance, they have the potential to aid in risk stratification and early detection of those who are at high risk of developing severe symptoms. This information can help to guide the allocation of healthcare resources, allowing for more intensive surveillance and focused therapies for patients who are regarded at high risk. Second, including these possible biomarkers in diagnostic tests has the potential to improve the accuracy of illness predictions. Third, the discovery of these biomarkers paves the way for the development of targeted medicines that modulate the linked pathways, with the goal of reducing disease severity and improving overall patient outcomes.

The study has some limitations. It focused on the analysis of transcriptome datasets from COVID-19 and diabetic peripheral neuropathic patients, which may not fully represent the entire population of individuals with these conditions. The study suggested potential drug molecules for the identified mutual differentially expressed genes (DEGs) based on comprehensive analysis, but further experimental validation is needed to confirm the effectiveness and safety of these potential drugs. It did not provide any characteristics of the patients included in the analysis, which may limit the generalizability of the findings. Lastly, the study did not discuss the potential confounding factors or limitations of the bioinformatics tools and databases used in the analysis.

## Conclusions

Our comprehensive analysis revealed that four common genes, B4GALNT2, MTX1, POLR2J, and TUBB4B are differentially expressed in patients with both COVID-19 and diabetic peripheral neuropathy. By examining these differentially expressed genes (DEGs), we identified shared pathways and GO functions across biological, molecular, and cellular contexts. Furthermore, we explored their intricate networks with other genes and regulatory mechanisms. Based on these findings, we propose potential drug candidates for treating patients with COVID-19 and diabetic peripheral neuropathy. However, experimental validation remains essential to confirm their efficacy.

## References

[1] (2020). The species severe acute respiratory syndrome-related coronavirus: classifying 2019-nCoV and naming it SARS-CoV-2. Nat Microbiol.

[2] Walls AC, Park YJ, Tortorici MA, Wall A, McGuire AT, Veesler D (2020). Structure, function, and antigenicity of the SARS-CoV-2 spike glycoprotein. Cell.

[3] Al Zamane S, Nobel FA, Jebin RA (2021). Development of an in silico multi-epitope vaccine against SARS-COV-2 by précised immune-informatics approaches. Inform Med Unlocked.

[4] Horn E, Chakinala MM, Oudiz R, Joseloff E, Rosenzweig EB (2020). Author rebuttal to response regarding "Letter to the Editor regarding 'Could pulmonary arterial hypertension patients be at lower risk from severe COVID-19?'". Pulm Circ.

[5] Yang X, Yu Y, Xu J (2020). Clinical course and outcomes of critically ill patients with SARS-CoV-2 pneumonia in Wuhan, China: a single-centered, retrospective, observational study. Lancet Respir Med.

[6] Wang X, Liu Z, Li J (2020). Impacts of Type 2 Diabetes on Disease Severity, Therapeutic Effect, and Mortality of Patients With COVID-19. J Clin Endocrinol Metab.

[7] Lim S, Bae JH, Kwon HS, Nauck MA (2021). COVID-19 and diabetes mellitus: from pathophysiology to clinical management. Nat Rev Endocrinol.

[8] Venmans LM, Bont J, Gorter KJ, Verheij TJ, Rutten GE, Hak E (2008). Prediction of complicated lower respiratory tract infections in older patients with diabetes. Br J Gen Pract.

[9] Barrett T, Wilhite SE, Ledoux P (2013). NCBI GEO: archive for functional genomics data sets--update. Nucleic Acids Res.

[10] Blanco-Melo D, Nilsson-Payant BE, Liu WC (2020). Imbalanced host response to SARS-CoV-2 drives development of COVID-19. Cell.

[11] Luo L, Zhou WH, Cai JJ (2017). Gene expression profiling identifies downregulation of the neurotrophin-MAPK signaling pathway in female diabetic peripheral neuropathy patients. J Diabetes Res.

[12] Gentleman R, Carey VJ, Huber W, Irizarry RA, Dudoit S (2005). Bioinformatics and computational biology solutions using R and Bioconductor. New York: Springer.

[13] Subramanian A, Kuehn H, Gould J, Tamayo P, Mesirov JP (2007). GSEA-P: a desktop application for Gene Set Enrichment Analysis. Bioinformatics.

[14] Kuleshov MV, Jones MR, Rouillard AD (2016). Enrichr: a comprehensive gene set enrichment analysis web server 2016 update. Nucleic Acids Res.

[15] Slenter DN, Kutmon M, Hanspers K (2018). WikiPathways: a multifaceted pathway database bridging metabolomics to other omics research. Nucleic Acids Res.

[16] Kanehisa M, Goto S (2000). KEGG: kyoto encyclopedia of genes and genomes. Nucleic Acids Res.

[17] Huang R, Grishagin I, Wang Y (2019). The NCATS BioPlanet - an integrated platform for exploring the universe of cellular signaling pathways for toxicology, systems biology, and chemical genomics. Front Pharmacol.

[18] Fabregat A, Jupe S, Matthews L (2018). The reactome pathway knowledgebase. Nucleic Acids Res.

[19] Pico AR, Kelder T, van Iersel MP, Hanspers K, Conklin BR, Evelo C (2008). WikiPathways: pathway editing for the people. PLoS Biol.

[20] Kanehisa M (2019). Toward understanding the origin and evolution of cellular organisms. Protein Sci.

[21] Gillespie M, Jassal B, Stephan R (2022). The reactome pathway knowledgebase 2022. Nucleic Acids Res.

[22] Szklarczyk D, Gable AL, Lyon D (2019). STRING v11: protein-protein association networks with increased coverage, supporting functional discovery in genome-wide experimental datasets. Nucleic Acids Res.

[23] Ewing RM, Chu P, Elisma F (2007). Large-scale mapping of human protein-protein interactions by mass spectrometry. Mol Syst Biol.

[24] Ben-Hur A, Noble WS (2005). Kernel methods for predicting protein-protein interactions. Bioinformatics.

[25] Szklarczyk D, Franceschini A, Wyder S (2015). STRING v10: protein-protein interaction networks, integrated over the tree of life. Nucleic Acids Res.

[26] Shannon P, Markiel A, Ozier O (2003). Cytoscape: a software environment for integrated models of biomolecular interaction networks. Genome Res.

[27] Mahmud SM, Al-Mustanjid M, Akter F (2021). Bioinformatics and system biology approach to identify the influences of SARS-CoV-2 infections to idiopathic pulmonary fibrosis and chronic obstructive pulmonary disease patients. Brief Bioinform.

[28] Odriozola A, Ortega L, Martinez L (2021). Widespread sensory neuropathy in diabetic patients hospitalized with severe COVID-19 infection. Diabetes Res Clin Pract.

[29] Babu G, Nobel FA (2022). Identification of differentially expressed genes and their major pathways among the patient with COVID-19, cystic fibrosis, and chronic kidney disease. Inform Med Unlocked.

